# Novel compound heterozygous variants in the *CSPP1* gene causes Joubert syndrome: case report and literature review of the *CSPP1* gene’s pathogenic mechanism

**DOI:** 10.3389/fped.2024.1305754

**Published:** 2024-03-22

**Authors:** Caichuan Wei, Haiju Zhang, Miaoying Fu, Jingping Ye, Baozhen Yao

**Affiliations:** Department of Pediatrics, Renmin Hospital of Wuhan University, Wuhan, Hubei, China

**Keywords:** *CSPP1*, Joubert syndrome, pathogenic mechanism, developmental delay, microcephaly

## Abstract

Joubert syndrome (JS) is a rare autosomal recessive neurodevelopmental condition characterized by congenital mid-hindbrain abnormalities and a variety of clinical manifestations. This article describes a case of Joubert syndrome type 21 with microcephaly, seizures, developmental delay and language regression, caused by a *CSPP1* gene variant and examines the contributing variables. This paper advances the understanding of JS by summarizing the literature and offering detection patterns for practitioners with clinical suspicions of JS.

## 1 Introduction

Joubert syndrome (JS) is a rare neurological and developmental dysfunctional autosomal recessive disorder that primarily affects cerebellar and brainstem morphogenesis (1). It is characterized by delayed developmental milestones, ataxia, decreased muscle tone, abnormal respiratory patterns, and abnormal eye movements in infancy (2). Magnetic resonance imaging (MRI) of the brain reveals a distinctive mid-hindbrain malformation known as the “molar tooth sign (MTS)”, which is the key diagnostic feature of the disease (3). The condition was later named after the French neurologist Marie Joubert, who originally documented this clinical finding in 1969 (4). The current prevalence is estimated at 1:80,000–1:100,000, with variable incidence and severity depending on the presentation (5). The prognosis depends on the development of the cerebellar vermis and the severity of the respiratory disease after birth (6, 7).

Over 35 genes have been found to be altered in JS, accounting for up to 94% of cases (8). These genes all encode proteins involved in the formation of primary cilia and their appendages, which classifies Joubert syndrome as a ciliary disease (9–11). Cilia are quasi-universal microtubule-based sensory organelles that plays a critical role in signal transduction during development and cellular homeostasis (12). Dysfunctional cilia may result in a group of Mendelian diseases known as ciliopathies, which are diagnosed based on clinical phenotype and genetic causes, are caused by cilia dysfunction (13). Based on the major clinical manifestations, ciliopathies can be classified as motile or nonmotile (14). Furthermore, primary ciliary dyskinesia, for instance, is characterized by severe respiratory problems and situs defects (15). Human ciliopathies such as Joubert syndrome, Bardet-Biedlsyndrome (BBS), and Meckel-Gruber syndrome manifest with defective primary cilia (16).

This report describes a patient with Joubert syndrome type 21 who presented with developmental delay, language regression, seizures, and microcephaly due to novel compound heterozygous variants in the centrosome and spindle pole associated protein 1 (CSPP1) gene on chromosome 8q13. Considering the rarity of Joubert syndrome and the significant phenotypic variability and genetic heterogeneity, this case study and a review of the pertinent literature will aid clinicians in better understanding JS, assisting in its early detection and diagnosis.

## 2 Case description

**Figure 1:**
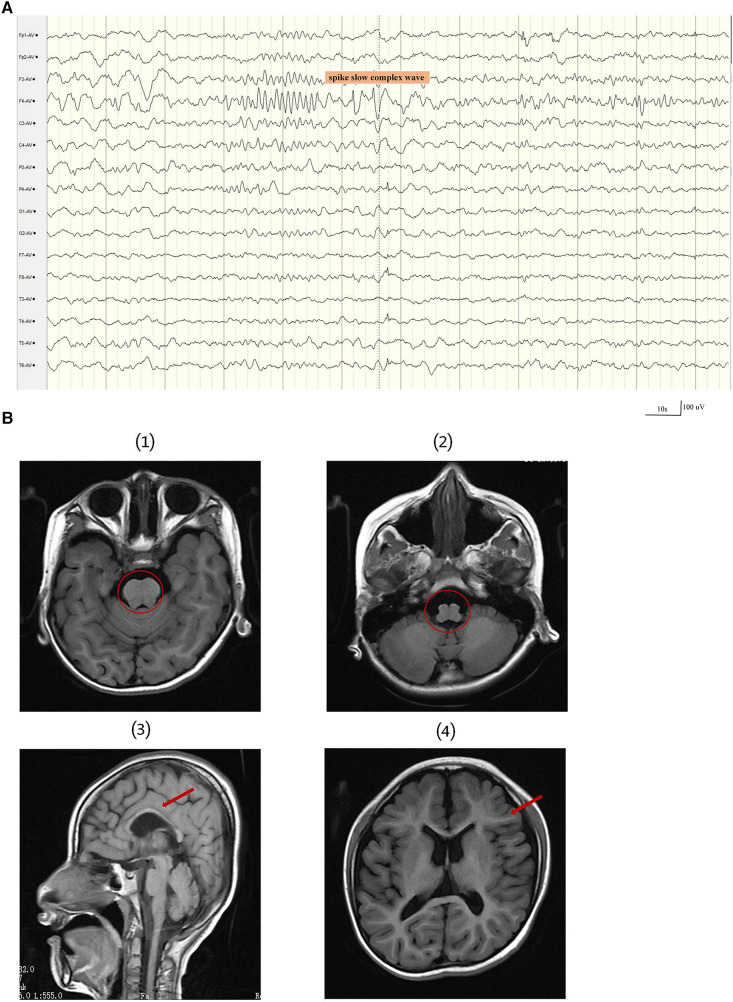
A 12 h sleep EEG clip and brain MRI results of the patient. (**A**) The EEG revealed medium-amplitude spikes and spike slow complex wave in the right frontal area. EEG signals were filtered using a 60 Hz notch filter, 6 Hz high-pass filter and 35 Hz low-pass filter. (**B**) The first and second images show the molar tooth sign (red circle). (3) The third image displays thin corpus callosum. (4) The fourth image shows a small white matter volume.

At 34 weeks and 2 days, the baby was delivered by cesarean section and weighed 2.1 kg at birth. He first raised his head at the age of 6 months, had tooth eruption at the age of seven months, could sit at 18 months of age, and began walking at the age of five. At the age of 2 years, a first epileptic seizure occurred. During the seizures, the patient exhibited a loss of consciousness, upward gaze, and generalized tonic-clonic activity. The patient experienced 3–4 such seizures per year, each lasting 2–5 min, but he could recover spontaneously. According to ILAE 2017 seizure classification (17), the seizure types were generalized tonic–clonic seizure. The patient had not received any anti-seizure medicines prior to seeking treatment at our clinic. The patient was 7 years and 6 months at his first visit to our clinic, showing cognitive impairment. At that time, he only understood simple instructions, had a limited vocabulary, and could only say a few simple words, like “bye-bye”. The patient's language expression and vocabulary are much reduced than before. The head circumference was 45 cm, with abnormal palmprint and wide-based gait. The 12 h sleep Electroencephalogram(EEG) revealed medium-amplitude spikes in the right frontal area and spike slow complex wave of about 3 Hz extending to the ipsilateral temporalis (Figure 1A). The molar tooth sign was observed on brain MRI, which is a critical imaging characteristic of Joubert syndrome. The brain MRI results also include hypoplastic of corpus callosum, ventricular enlargement, widening of the cerebral sulcus, Small volume of cerebral white matter and Cranial volume is less than the facial cranium (Figure 1B). The patient's medical history and brain MRI suggested hereditary causes, so a genetic test was recommended. Written informed consent was obtained from the parents before the patient and parents underwent trio-whole-exome sequencing (trio-WES).

### 2.1 Trio-whole-exome sequencing and sanger sequencing

Sample testing was performed at the AEGICARE medical testing laboratory in shenzhen, china. Genomic DNA was extracted from peripheral blood samples using the QIAamp Blood Genomic DNA Extraction Kit (Cat No./ID: 51104), and the concentration and purity of DNA were measured by NanoDrop2000. DNA was fragmented to a length range of 150–300 bp using a Covaris M220 DNA sonicator. The fragmented DNA was amplified by end-repair and plus-A reactions, ligating the junctions. Targeted capture of amplified libraries was performed using the xGen® Exome Research Panel v1.0 kit (IDT). PE150 sequencing was performed using Illumina Novaseq 6000.

For the bioinformatics analysis, reads were aligned to the human reference genome (GRCH37/hg19) using Burrows-Wheeler-Alignment Tool (BWA), and single nucleotide polymorphisms(SNPs), insertion and deletion (indel) were detected using the genome analysis toolkit (GATK) standard variant detection process. Subsequently, the results of the obtained variants were annotated using ANNOVAR. During the screening of variant loci, variants with allele frequencies greater than 1% in the gnomAD, ExAC, Thousands database, and ESP6500 databases were filtered out. Candidate genes are then prioritised to look into potentially deleterious variations. According to the ACMG (American College of Medical Genetics and Genomics) genetic variation classification criteria and guidelines, variation loci were also assessed for pathogenicity.

**Figure 2:**
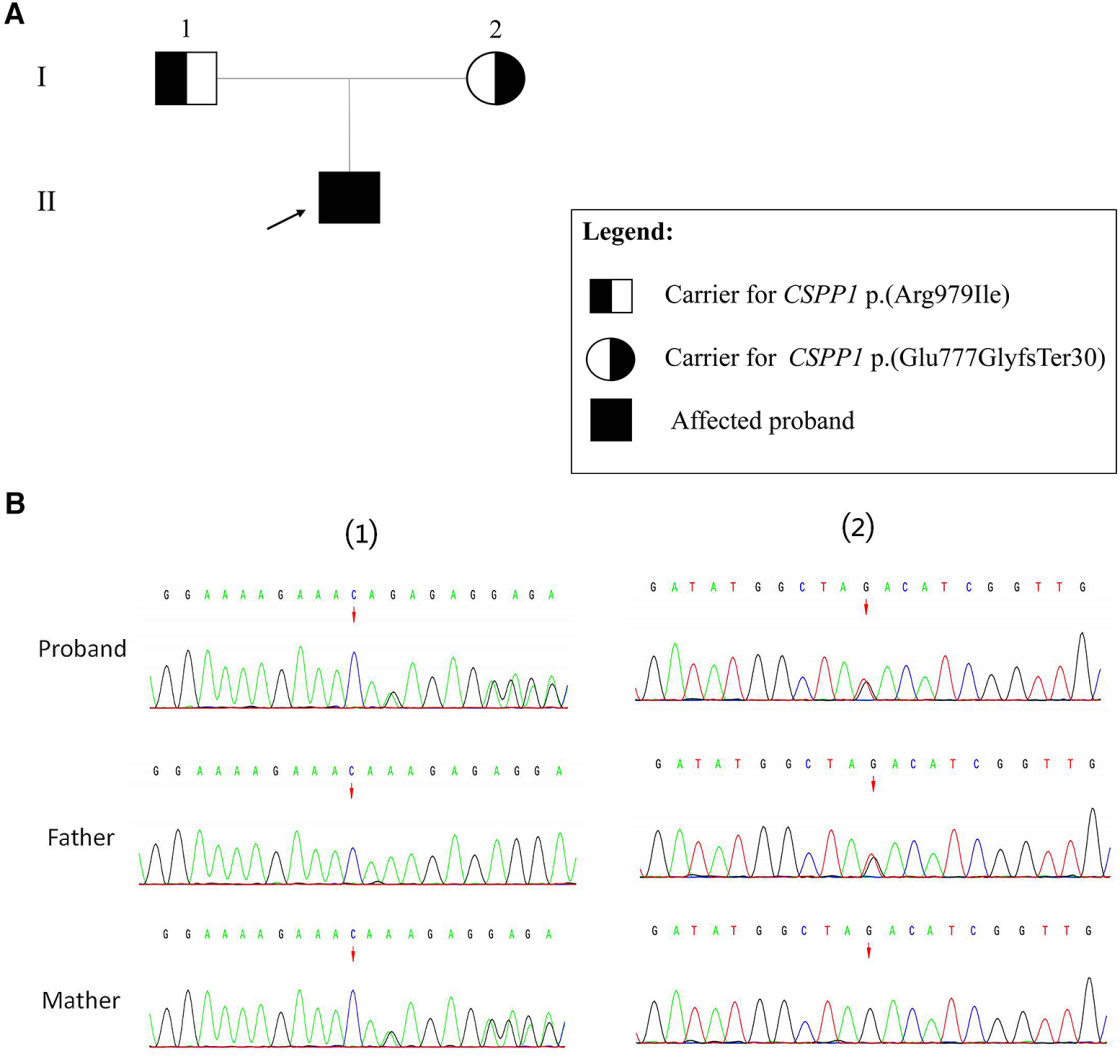
Sanger validation results. (**A**) Family pedigree showing segregation of *CSPP1* variants. Individuals carrying the heterozygous variants are indicated as black half-filled circles (females) or squares (males). The filled black symbols represent the affected members, and the arrow denotes the proband. (**B**) (1) The variant sequence of the *CSPP1* gene c.2325_2326del (p.Glu777GlyfsTer30) in the proband. Arrows indicate the variant site. (2) The variant sequence of the *CSPP1* gene c.2936G>T (p.Arg979Ile) in the proband. Arrows indicate the variant site.

The Trio-WES results revealed compound heterozygous variants in the *CSPP1* gene, namely c.2325_2326del (p.Glu777GlyfsTer30) and c.2936G>T (p.Arg979Ile). According to the ACMG variant classification criteria, the c.2325_2326del variant is classified as likely pathogenic, and the missense variant c.2936G>T is classified as a VUS (variant of unknown significance). Based on the family segregation analysis performed using sangers sequencing, the c.2325_2326del variant was inherited maternally, and the c.2936G>T variant was inherited paternally (Figure 2).

During hospitalization, we recommend that patients take anti-seizure medicine (valproic acid) and receive language and motor abilities training. After 1 month and 3 months of discharge, we conducted telephone follow-up and found that the patient had significant improvement in speech and motor skills. However, due to concerns surrounding the potential negative consequences of medication, parents autonomously ceased the administration of anti-epileptic treatment, thereby making it impractical to determine the efficacy of the medication.

## 3 Discussion

Joubert syndrome is an autosomal recessive congenital disease caused by a homozygote or compound heterozygous variant. According to previous literature (18–23), the affected children may exhibit neonatal hypotonia, abnormal eye movements, developmental delays, episodic respiratory dysregulation, progressive cerebellar ataxia development, and cognitive impairment. JS patients present with multisystem organ involvement, including fibrocystic kidney and liver disease, retinal dystrophy, chorioretinal coloboma, occipital encephalocele, and polydactyly (24). Some JS patients only exhibit neurological problems. This patient's brain MRI revealed the molar tooth sign, a characteristic cerebellum and brainstem abnormality characterized by a deep interpeduncular fossa and enlarged superior cerebellar peduncles; this sign is currently regarded as an indicator for the clinical diagnosis of JS (25). MTS also varies in severity, with minor cases being challenging to identify and more likely to go unnoticed. In addition to clinical presentation, JS also exhibits high genetic heterogeneity and a wide variety of conclusive and broadly applicable clinical genotype-phenotype correlations. However, a report (26) suggested that *CC2D2A* biallelic variants appear to be more frequently associated with seizures, while ocular involvement is often linked with *CEP290* and *AHI1* alterations. In addition, abnormal eye movements and strabismus tend to arise from *AHI1* molecular defects or *TMEM237* gene alterations; ptosis is most frequently associated with *TMEM67, CPLANE1,* and *CSPP1* defects; kidney disease is most often associated with *CEP290* and *TMEM6* defects. Moreover, a strong association was found between liver disease and the *TMEM67* gene.

In this report, we present a new case characterised by microcephaly, seizures, developmental delay, and language regression, which were revealed to be *CSPP1* gene variants on trio-WES. Brain MRI revealed the classic “molar tooth sign”, indicating that the new compound heterozygous variants in the *CSPP1* gene was the likely cause of Joubert syndrome. The patient mainly presented CNS symptoms without other organ symptoms, which indicates that the *CSPP1* gene variants are primarily linked to the classic type of JS. In 2014, three cases of *CSPP1* gene variants causing Joubert syndrome were reported (27–29). We analyzed these three papers based on patients’ clinical characteristics and the novel variants (Table 1). All affected people displayed a wide spectrum of clinical phenotypic severity, with the most severe patients exhibiting severe developmental disorders, occipital brain expansion, cleft palate, and skeletal changes. In contrast, the mildest patients only displayed mild developmental disorders without retinal, renal, or hepatic involvement. Cranial structural abnormalities, seizures, and renal lesions were infrequently noted; hypotonia and developmental delay were present in nearly patients, except those who had stillbirths. In our case, the new variant resulted in cranial abnormalities, seizures, and a decline in intelligence, which are uncommon clinical features of *CSPP1* variants. Therefore, this case report broadens the phenotypic spectrum of Joubert syndrome caused by *CSPP1* variants. All 31 patients, including our patient, displayed the recognizable “ molar teeth sign “ on brain MRI. Clinical suspicion of Joubert syndrome is warranted when encountering this radiological feature and additional precise investigations should be carried out to confirm the diagnosis. Nonetheless, the cause of the high phenotypic heterogeneity of *CSPP1* gene variants remains unclear. Changes in protein isoforms with various functions may result in variants in the same gene, leading to different diseases. The kind of allele, the existence of modifiers in various genetic backgrounds, the overlap of protein functions, and cell type specificity can all impact the phenotypic outcome (30). Based on the literature analysis and our patient information, JS presents with significant phenotypic heterogeneity, which hinders clinical recognition and diagnosis. However, RaeLynn-Forsyth et al. recently published a systematic analysis of 94 patients with Joubert syndrome and reported recognizable facial features in JS patients (20), which may assist clinical frontline workers in identifying JS more quickly. Children usually have triangular open mouths and prominent tongues, whereas adults demonstrate anterior jaw protrusion and prominent nasal bridge. In addition to these facial features, together with neurodevelopmental delay, cranial malformation, head tilt when looking straight ahead, abnormal eye movements, renal and/or liver disease, and polydactyly, might suggest JS. There is no specific treatment for Joubert syndrome (31, 32), but rehabilitation may improve movement and speech impairment. Regular blood biochemistry tests, eye examinations, and ultrasound examinations of the liver, kidney, and other vital organs are also recommended for early detection and treatment of problems.

**Table 1:** Clinical features and brain MRI of individuals with CSPP1 mutations.

First author, year	Individual	Age	cDNA mutation(allele)	Clinical phenotypes									Brain MRI	
				Cranial malformation	Seizures	Hypotonia	Delayed development	Breathing abnormalities	Abnormal eye movements	Retina	Kidney	Other	Molar tooth sign	Other
Akizu et al. (27)	1	unk	c.652C>T(homozygous)	−	−	+	+	−	+	+	unk	Bilateral ptosis	+	Cerebellar vermis dysgenesis, hypoplasia of the brainstem, thin corpus callosum
	2	unk	c.[950+1G>C];[3,205+1G>A] (compound heterozygous)	−	−	+	+	+	+	−	unk	−	+	Cerebellar vermis dysgenesis, hypoplasia of the brainstem
	3	unk	c.2,243_2244delAA(homozygous)	−	−	+	+	+	−	−	unk	−	+	Cerebellar vermis dysgenesis
	4	unk	c.[2260C>T];[457delA](compound heterozygous)	−	−	+	+	−	−	−	unk	Bilateral ptosis	+	Cerebellar vermis dysgenesis, hypoplasia of the brainstem, thin corpus callosum
	5	unk	c.2773C>T(homozygous)	−	−	+	+	+	+	−	unk	Bilateral ptosis and exotropia	+	Cerebellar vermis dysgenesis, thin corpus callosum
	6	unk	c.448C>T (homozygous)	−	−	+	+	+	+	−	unk	Bilateral ptosis	+	Cerebellar vermis dysgenesis
Shaheen et al. (28)	7	26 weeks	c.2244_2247del (homozygous)	−	−	−	−	−	−	−	Hyperechogenic	Single nostril, anophthalmia	−	Hydranencephaly, occipital encephalocel
	8	18 weeks	c.2244_2247del (homozygous)	−	−	−	−	−	−	−	Hyperechogenic	Single nostril, partially fused eyes	−	occipital encephalocele
	9	7 years	c.363_364delTA(homozygous)	−	−	+	+	−	−	−	−	strabismus	+	−
	10	28 days	c.363_364delTA (homozygous)	−	−	−	−	−	−	−	−	−	+	posterior fossa cyst
	11	24 days	c.363_364delTA (homozygous)	−	−	−	−	−	−	−	−	−	+	posterior fossa cyst
Tuz et al. (29)	12	1 year	c.2320C>T (homozygous)	+	−	+	+	+	−	−	−	JATD, MV, hypertelorism, NG tube, megalopapillae, optic atrophy	+	−
	13	12 years	c.2708delA (homozygous)	−	−	+	+	+	+	−	−	−	+	−
	14	4 years	c.[2244_2245delAA ];[2280delA](compound heterozygous)	−	−	+	+	+	+	+	−	JATD, G-tube, third-nerve palsy	+	−
	15	4 mouths	c.[2244_2245delAA];[2280delA](compound heterozygous)	−	−	+	+	+	+	−	+	JATD, MV, G-tube, dysphagia	+	−
	16	10 years	c.[3211_3212insA]; [2953+1G>A](compound heterozygous)	−	−	+	+	+	+	−	−	−	+	−
Tuz et al. (29)	17	14 years	c.[3211_3212insA]; [2953+1G>A](compound heterozygous)	−	−	+	+	+	+	−	−	−	+	−
	18	unk	c.2448_2454dupAGAAGAA(homozygous)	unk	unk	unk	unk	unk	unk	unk	unk	−	+	−
	19	2 years	c.[457delA];[2260C>T](compound heterozygous)	−	−	+	+	+	−	−	−	HL	+	unk
	20	21 years	c.[658C>T];[2527_2528delAT](compound heterozygous)	−	−	+	+	−	−	−	−	Liver fibrosis on biopsy, splenomegaly elevated transaminases, chronic sinusit	+	−
	21	16 years	c.[658C>T];[2527_2528delAT](compound heterozygous)	−	−	+	+	−	−	−	−	elevated transaminases, chronic sinusitis	+	−
	22	6 years	c.2527_2528delAT(homozygous)	−	−	+	+	+	−	−	−	Cleft palate, c hypertelorism	+	−
	23	22 years	c.[3205+1G>A];[950+1G>C](compound heterozygous)	−	−	+	+	+	+	+	−	High-arched palate	+	−
	24	16 years	c.[652C>T];[1214G>A](compound heterozygous)	−	−	+	+	+	+	−	−	Obese	+	−
	25	9 years	c.[652C>T];[1214G>A](compound heterozygous)	−	−	+	+	−	+	−	−	−	+	−
	26	5 years	c.1132C>T; c.2441_2444delAGAA(compound heterozygous)	−	−	+	+	+	−	−	unk	Hypertelorism, CPEO,mild left optic disc pallor	+	−
	27	2 years	c.1132C>T; c.2441_2444delAGAA(compound heterozygous)	−	−	+	+	−	−	−	unk	Hypertelorism, restricted upward gaz	+	−
	28	7 mouths	c.2527_2528delAT(homozygous)	−	−	+	+	+	−	−	−	JATD, NG tube, HL, high-arched palat	+	−
	29	9 years	c.3211_3212insA;c.2953+1G>A(compound heterozygous)	−	−	+	+	+	+	−	−	−	+	−
	30	6 years	c.2448_2454delAGAAGAA (homozygous)	−	−	+	+	−	−	−	−	−	+	−
Present study	31	7.6 years	c.[2325_2326del];[2936G>T](compound heterozygous)	+	+	+	+	−	−	−	−	Regressed in development	+	Hypoplastic corpus callosum, a smaller white matter volume

unk, unknown; ATD, jeune asphyxiating thoracic dystrophy; MV, mechanical ventilation; NG tube, nasogastric tube; HL, hearing loss; G-tube, gastrostomy tube; Individuals 26, 27, and 29 were not specifically mentioned as sources of variants in the original reference.

Despite the ample clinical evidence to supporting the association between the *CSPP1* gene and Joubert syndrome, few studies have explored the disease's pathogenesis. Previous research was analyzed to explore the mechanism of the *CSPP1* gene causing JS. Centrosome and spindle pole associated protein 1 (CSPP1) was initially found at centrosomes and mitotic spindle poles, but only later showed to be localized to primary cilia (33). The protein is encoded by the *CSPP1* gene, which was initially found to be overexpressed in B-cell lymphomas (27). CSPP1 binds to the centrosome and microtubule; it is localized at the central spindle in mid-life and at the central groove in late life and during cell division. It plays an essential role in cell cycle-dependent cytoskeletal structure and the development of cilia (34). The widespread expression of *CSPP1* in neural tissues, especially in the growing cerebellum, is directly related to the specific mid-hindbrain deformity seen in children with *CSPP1* variants (27). The primary cilia play an essential role in the SHH, PDGF, and WNT signaling cascades, which are crucial to neuronal development and patterning (35). Studies have demonstrated that impaired ciliary Hedgehog (Hh) signaling results in the phenotype associated with JS (36). Hedgehog (Hh) is one of the few signaling pathways that is repeatedly used for intercellular communication during development (37). Three mammalian Hh proteins have been discovered, namely Sonic (SHH), Indian (IHH), and Desert (DHH). Shh is essential for the creation of the central nervous system, face structure, and limbs (38). IHH is involved in skeletal development, and DHH plays a role in gonadal development (39). Vertebrate Hh signaling is solely dependent on primary cilia, whereas CSPP-L, a subtype of CSPP1*,* interacts with CEP104 to create cilia with Hh signaling capability (36). Moreover, *CSPP1* dysfunction seems to have an impact on the growth, length, and transportation of main ciliary trichomes to axons (40). Primary cilia are about 1/1000th the size of the cytosol, and the absence of adequate primary cilia signaling results in altered axonal growth cone dynamics and filopodia-to-thin pseudopod balance. These changes lead to defective axonal development and impair neuronal connections in the mammalian brain, potentially causing MTS, a specific axonal malformation (30). Primary cilia are ubiquitous and defects can adversely affect multiple organ systems.

## 4 Conclusion

This paper presents the case of a patient with Joubert syndrome caused by *CSPP1* gene variation, manifesting developmental delay, language regression, seizures, and microcephaly. Furthermore, the contributing factors and prior case reports of the *CSPP1* gene variant were listed and assessed, discussing the high clinical variability and genetic heterogeneity associated with Joubert syndrome. By reporting this case report, more information can be gained about the clinical features and pathogenesis of this rare disease.

## Data Availability

The original contributions presented in the study are included in the article/Supplementary Material, further inquiries can be directed to the corresponding authors.
