# Supplementary figures and images for: Novel compound heterozygous variants in the CSPP1 gene causes Joubert syndrome: case report and literature review of the CSPP1 gene’s pathogenic mechanism
Source: Front Pediatr. 2024 Mar 22;12:1305754. doi: 10.3389/fped.2024.1305754 (PMC10995352; doi:10.3389/fped.2024.1305754)

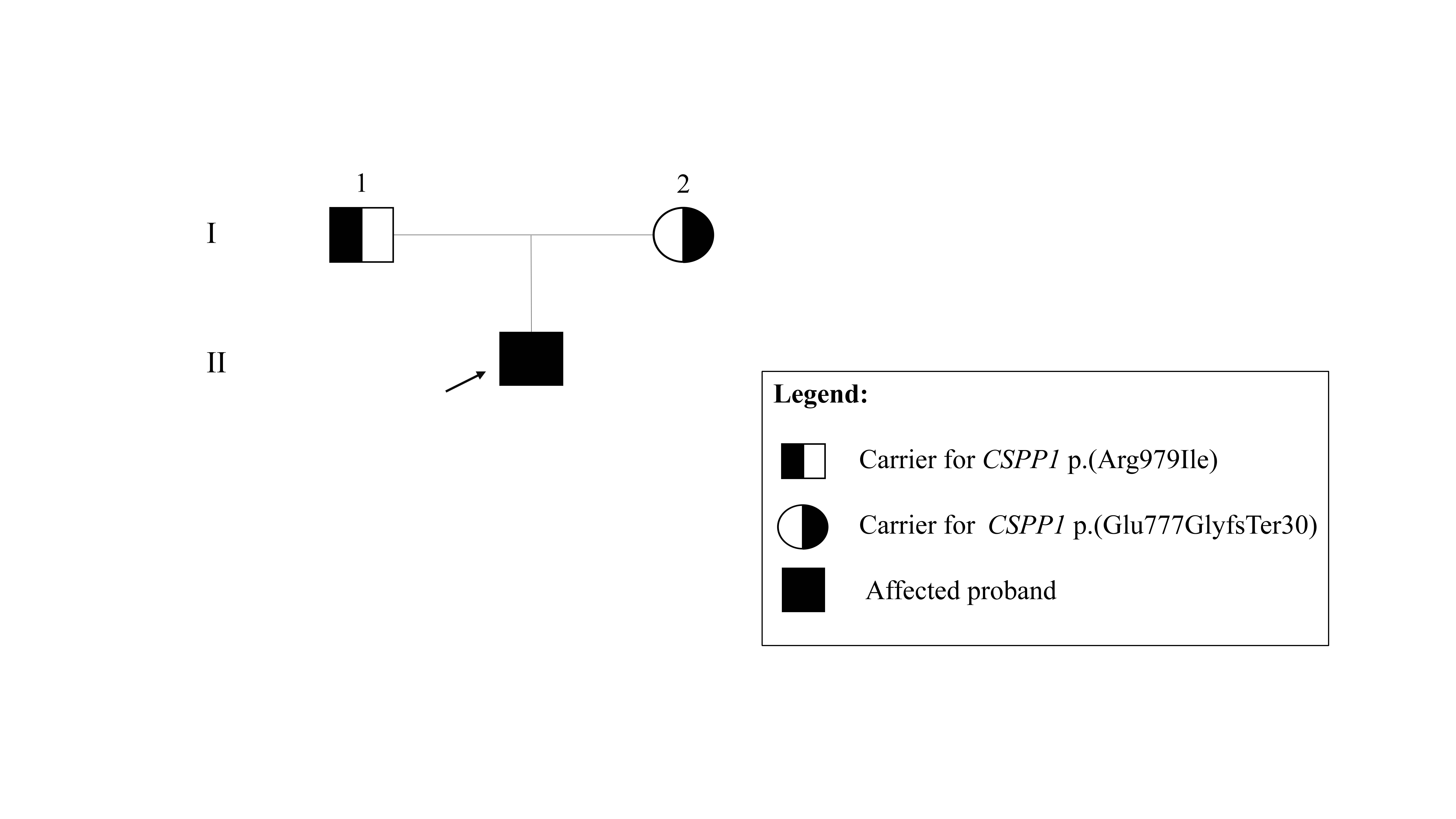

Supplement: Supplementary file 1 [file Image1.jpeg]
